# Na^+^/H^+^ Exchangers (NHEs) in Mammalian Sperm: Essential Contributors to Male Fertility

**DOI:** 10.3390/ijms241914981

**Published:** 2023-10-07

**Authors:** Cameron C. Gardner, Paul F. James

**Affiliations:** Department of Biology, Miami University, Oxford, OH 45056, USA; gardnecc@miamioh.edu

**Keywords:** Na^+^/H^+^ exchangers (NHEs), SLC9, sperm, pH regulation, male fertility, motility, capacitation, acrosome reaction, fertilization

## Abstract

Na^+^/H^+^ exchangers (NHEs) are known to be important regulators of pH in multiple intracellular compartments of eukaryotic cells. Sperm function is especially dependent on changes in pH and thus it has been postulated that NHEs play important roles in regulating the intracellular pH of these cells. For example, in order to achieve fertilization, mature sperm must maintain a basal pH in the male reproductive tract and then alkalize in response to specific signals in the female reproductive tract during the capacitation process. Eight NHE isoforms are expressed in mammalian testis/sperm: NHE1, NHE3, NHE5, NHE8, NHA1, NHA2, NHE10, and NHE11. These NHE isoforms are expressed at varying times during spermatogenesis and localize to different subcellular structures in developing and mature sperm where they contribute to multiple aspects of sperm physiology and male fertility including proper sperm development/morphogenesis, motility, capacitation, and the acrosome reaction. Previous work has provided evidence for NHE3, NHE8, NHA1, NHA2, and NHE10 being critical for male fertility in mice and NHE10 has recently been shown to be essential for male fertility in humans. In this article we review what is known about each NHE isoform expressed in mammalian sperm and discuss the physiological significance of each NHE isoform with respect to male fertility.

## 1. Introduction

The Na^+^/H^+^ exchangers (NHEs) are a branch of the cation/proton antiporter (CPA) superfamily of transporters that are found in the membranes of many cells. NHEs found on the plasma membrane of higher eukaryotic cells export intracellular protons (H^+^) using the energy stored in the inward directed sodium ion (Na^+^) electrochemical gradient established by the Na,K-ATPase [1]. By exporting protons, NHEs play an important role as regulators of intracellular pH in many cells [2,3,4,5,6] and thus affect a wide variety of cellular and physiological processes from brain function [7] to male fertility [8,9,10,11,12,13,14,15]. In addition to those found on the plasma membrane, other NHEs display a predominantly intracellular localization and are thought to play roles in regulating pH in intracellular compartments [16].

After ejaculation, sperm must undergo a maturation process in the female reproductive tract, known as capacitation, before they are able to bind to and fertilize an oocyte [17]. There are many signature molecular events that occur during capacitation, including: an increase in intracellular pH [2,18,19,20,21], increases in intracellular cyclic adenosine monophosphate (cAMP) levels resulting in increased protein tyrosine phosphorylation [22,23,24], increased K^+^ permeability [25,26,27], hyperpolarization of the sperm plasma membrane [25,26,28,29], and an increase in intracellular Ca^2+^ [25,26,30,31]. Capacitation ultimately results in sperm hyperactivation and the ability to undergo the acrosome reaction which are events necessary for the sperm to be able to fertilize an oocyte.

Intracellular pH (pH_i_) has long been known to be important for a wide range of physiological processes including sperm motility [18,32,33] as pH likely affects multiple proteins vital for sperm function such as the Ca^2+^ channel complex CatSper (cation channel of sperm) and the K^+^ channel SLO3 (one of the vertebrate homologs of the Drosophila slowpoke protein) [25,26,30,34,35]. Studies have shown that the transition from the immotile to the motile state and/or from a less motile to a highly motile state of rat and bovine sperm is mediated through a rise in intracellular pH [2,18,19,20,21]. In fact, sperm pH_i_ positively correlates with both hyperactivated motility and successful in vitro fertilization (IVF) in normospermic human patients [36]. Moreover, it has been demonstrated that the outer dynein arms of human sperm contain a pH sensitive regulatory mechanism whereby a slight alkalization of the cytoplasm initiates and increases the enzymatic activity of dynein motor proteins and thus modulates flagellar motion [37].

There is ample evidence suggesting that NHE activity plays an important role in the regulation of intracellular pH and motility of sperm. For example, it has been suggested that the alkalization required for the initiation of motility in sea urchin sperm is mediated by NHE activity [33,38,39]. It has also been shown that amiloride, an inhibitor of NHEs, inhibits rat sperm motility suggesting the importance of Na^+^ influx and/or H^+^ efflux in mammalian sperm motility [2]. Furthermore, in human sperm, inhibition of NHE activity with a more specific NHE inhibitor, 5-*N*-ethyl-*N*-isopropyl amiloride (EIPA), or incubation in Na^+^ deficient media, results in the acidification of the cytoplasm in capacitated human sperm suggesting the importance of NHE activity in the regulation of sperm pH_i_ in humans [40,41]. Inhibition of NHE activity with another NHE-specific inhibitor, 5-(*N*,*N*-dimethyl)-amiloride (DMA), results in impaired CatSper and Slo3 activity in mouse sperm [35]. Finally, targeted genetic inactivation of certain NHE genes in mice has been shown to negatively impact sperm motility and male fertility [8,9,10,11,42] and a recent clinical study found that a mutation in an NHE-encoding gene resulted in human male infertility [13].

**Figure 1:**
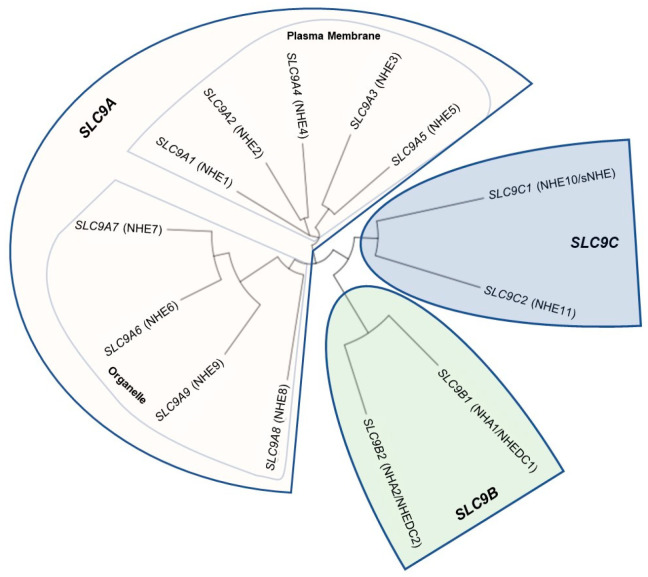
Phylogenetic tree of the solute carrier family 9 (*SLC9*) genes encoding human Na^+^/H^+^ Exchanger (NHE) proteins. Human protein sequences were aligned using Clustal Omega (https://www.ebi.ac.uk/Tools/msa/clustalo/, accessed on 7 October 2022) and the tree was generated using Interactive Tree of Life (https://itol.embl.de/ [49]). Protein sequence identifiers: *SLC9A1*, NP_003038.2; *SLC9A2*, NP_003039.2; *SLC9A3*, NP_004165.2; *SLC9A4*, NP_001011552.2; *SLC9A5*, NP_004585.1; *SLC9A6*, NP_001036002.1; *SLC9A7*, NP_001244220.1; *SLC9A8*, NP_001247420.1; *SLC9A9*, NP_775924.1; *SLC9B1*, NP_631912.3; *SLC9B2*, NP_849155.2; *SLC9C1*, NP_001307460.1; *SLC9C2*, NP_848622.2. The *SLC9A* subfamily is highlighted in pink and is further divided into *SLC9A1–5* because of their established plasma membrane localization and then *SLC9A6–9* which localize intracellularly (note that NHE3, NHE5, and NHE8 have reported both intracellular and plasma membrane localizations). The *SLC9B* subfamily is highlighted in green while the *SLC9C* subfamily is highlighted in blue. Reprinted/adapted from [43].

**Figure 2:**
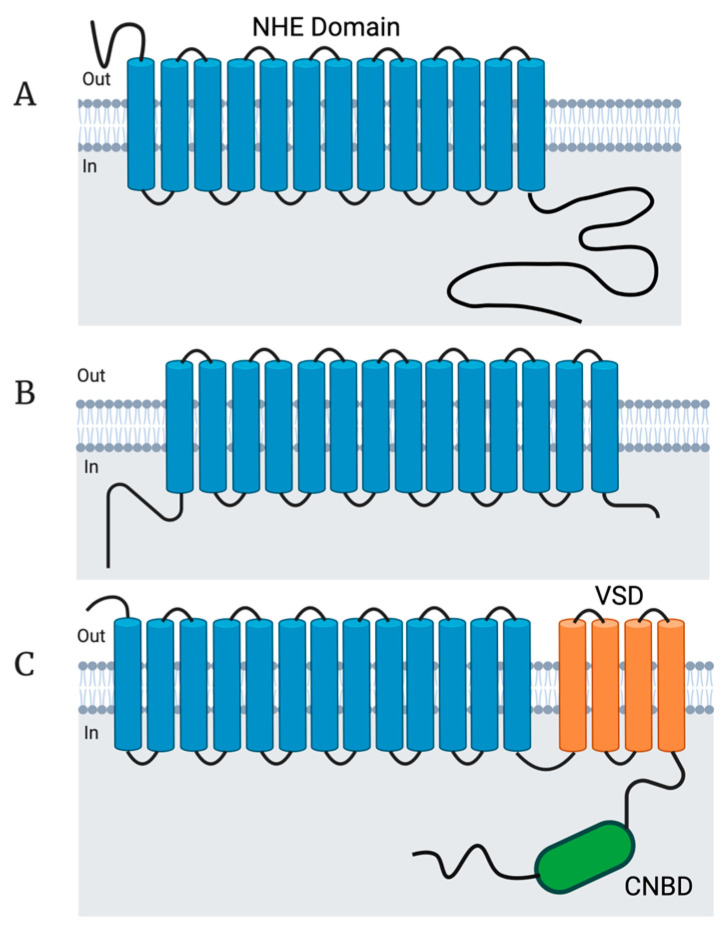
NHE structural features. (**A**) Topology of human NHE1. The structure of human NHE1 was solved and found to contain 13 transmembrane domains which comprise a conserved NHE domain [50]. The NHE1–9 isoforms are all predicted to contain an NHE domain within their transmembrane domains as well as short *N*-termini and long intracellular C-termini of varying lengths and functions. NHE1 is known to have multiple regulatory domains in the intracellular C-terminus which control different aspects of pH sensing as well as the cell cycle. (**B**) Topology of bison NHA2. The structure of bison NHA2 was solved and found to possess 14 transmembrane domains. Both the NHA1 and NHA2 human isoforms are also predicted to contain an NHE domain within their 14 transmembrane domains as well as an intracellular *N*-terminus and a short C-terminus. (**C**) Predicted topology of human NHE10. Both human NHE10 and NHE11 are predicted to contain an NHE domain within the first 13 transmembrane domains, followed by 4 transmembrane domains comprising a voltage sensing domain (VSD; orange), followed by an intracellular cyclic nucleotide binding domain (CNBD; green). Topology figure created with Biorender.com.

Na^+^/H^+^ Exchanger proteins (NHEs) are encoded by the solute carrier family 9 (*SLC9*) gene family of solute carriers. This gene family has been divided into three subfamilies, determined by sequence homology: the NHE subfamily (*SLC9A1*-*SLC9A9* which encode the NHE1-NHE9 proteins), the NHA subfamily (*SLC9B1* and *SLC9B2* which encode the NHA1 and NHA2 proteins; also known as NHEDC1 and NHEDC2), and the mammalian sperm-NHE-like subfamily (*SLC9C1* and *SLC9C2* which encode the NHE10 (also known as sNHE) and NHE11 proteins) [5,6,43] (Figure 1). The NHE subfamily contain a conserved NHE domain at the *N*-terminus of the protein and also contain a cytosolic C-terminus of varying length that serves different regulatory functions for the nine different isoforms [6] (Figure 2). The NHE subfamily can be further divided by subcellular localization: NHE1, 2, 4 are known to localize to the plasma membrane, while NHE6, 7, and 9 are known to localize to membranes of intracellular organelles, while NHE3, NHE5, and NHE8 have been shown to localize to either the plasma membrane or intracellular organelles in certain tissues and conditions [5,6]. *SLC9B1* and *SLC9B2* cluster into their own subfamily because they show closer sequence homology to bacterial Na^+^/H^+^ antiporters (NHAs) than to mammalian NHEs [44] (Figure 2). Prokaryotic NHAs are electrogenic and transport Na^+^ out of the cell and H^+^ into the cell, driven by the H^+^ gradient established by H^+^-ATPases [1,45,46]. *SLC9C1* and *SLC9C2* appear to exist only in metazoans [47] and cluster into the mammalian sperm-NHE-like subfamily because of their unique predicted protein structure containing a NHE domain at the *N*-terminus, followed by a voltage-sensing domain (VSD), and an intracellular cyclic nucleotide binding domain (CNBD) at the C-terminus [6,8,44] (Figure 2). For a detailed analysis on NHE evolution and conserved residues important for ion transport, see [6,44,48].

**Figure 3:**
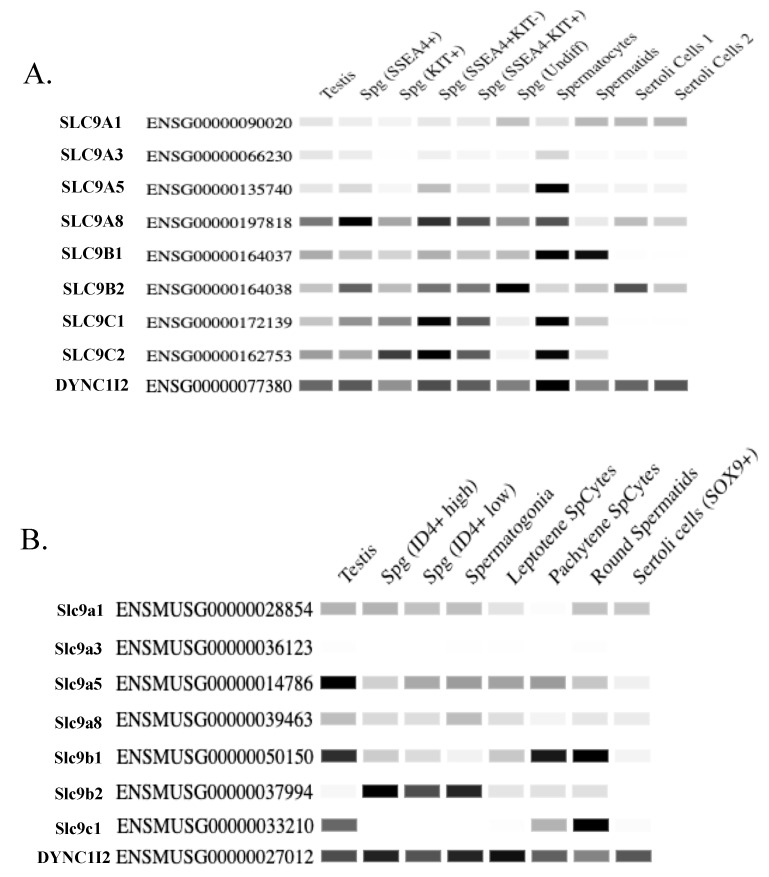
RNA sequencing data of *SLC9* genes expressed in testis/sperm. (**A**,**B**) Quantitative heatmaps depicted as conventional semi-quantitative PCR of *SLC9* genes expressed in testis/sperm displaying relative expression of these genes across different testis cell types in (**A**) human and (**B**) mice. *DYNC1I2* is included as a loading control in both the human and mouse heatmaps as this gene exhibits relatively consistent expression across the measured cell types [52]. *SSEA-4* is a marker for repopulating human spermatogonial stem cells [53], and *Kit* is a marker for differentiating spermatogonia [54,55]. Spermatogonia exhibiting high *ID4* expression are hallmarks of renewing spermatogonial stem cells, whereas lower *ID4* expression is associated with spermatogonia that are in transition to the progenitor state [56]. Note that *Slc9C2* is annotated as a pseudogene in mice and therefore there is no RNA-seq data for this gene in mice. This data is taken and the figures generated from the Mammalian Reproductive Genetics Database V2 (https://orit.research.bcm.edu/MRGDv2, accessed on 20 June 2023 [57]). Note that only *SLC9* genes for which there is published data of their protein expression in testis/sperm from the literature are included in this figure.

**Figure 4:**
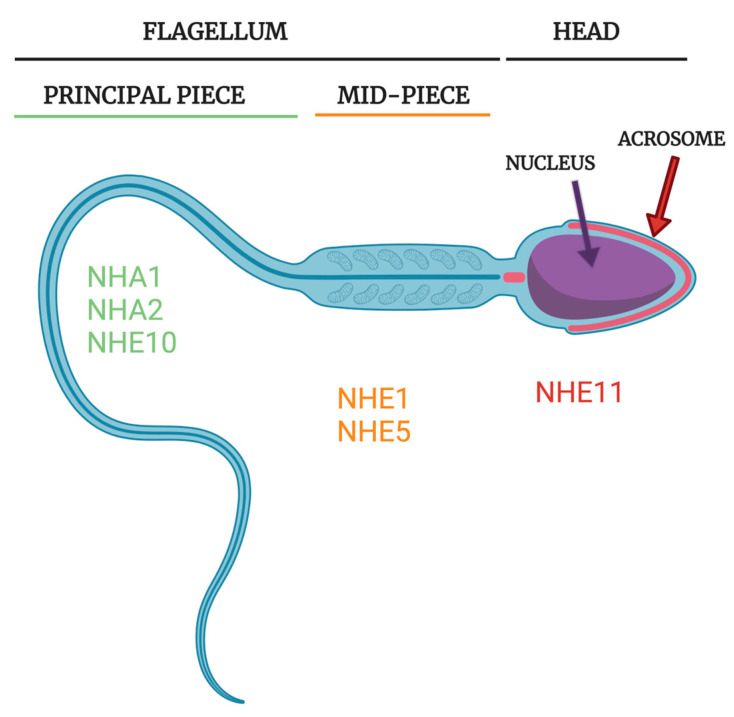
Subcellular NHE localization in rodent and human spermatozoa. NHE1 and NHE5 are known to localize to the midpiece in rat sperm [51], potentially playing a role in energy homeostasis or simply in basal pH regulation. NHA1 has been reported to localize to the principal piece in human and both the principal piece and midpiece in mouse sperm and NHA2 has been reported to localize to the principal piece in mouse sperm [10,11]. NHE10 has been reported to localize to the principal piece in mouse sperm [8]. In different publications, human NHE10 has been reported to localize to either the entire flagellum [13] or just the principal piece [12]. Localization of NHEs to the principal piece may help support sperm motility because intracellular pH regulation of the principal piece greatly influences sperm motility. NHE11 has recently been reported to localize specifically to the sperm head, likely the plasma membrane overlying the acrosome, in both rat and human sperm [43]. The exact role of NHE11 in the sperm head is unclear but could potentially regulate the acrosome reaction or sperm/egg fusion events. Note that neither NHE3 nor NHE8 proteins have yet to be demonstrated to be expressed in mature sperm cells. Sperm figure created with Biorender.com.

There are eight NHE protein isoforms known to be expressed in mammalian testis/sperm: NHE1, NHE3, NHE5, NHE8, NHA1, NHA2, NHE10, and NHE11 [8,9,10,42,43,51] (Figure 3 and Figure 4; Table 1). In this review, we will be examining the NHE proteins that are known to be expressed in sperm as well as discussing their transport activity and the role that they play in sperm physiology. We will attempt to describe the significance of individual NHEs in male fertility and posit potential implications for human medicine and society.

## 2. *SLC9C1* (NHE10/sNHE)

### 2.1. Molecular Genetics and Expression Patterns

*SLC9C1* is located on human Chromosome 3 and is comprised of 29 exons. Both the mouse and human NHE10 proteins are expected to contain 17 total transmembrane domains: the first thirteen transmembrane domains are predicted to encode an *N*-terminal NHE domain, and the last four transmembrane domains are predicted to encode a voltage sensing domain (VSD). The C-terminus of NHE10 is predicted to encode an intracellular cyclic nucleotide binding domain (CNBD) [8]. The predicted structure of NHE10 suggests that the NHE transport activity of the protein may be regulated by cyclic nucleotides and/or sperm membrane potential (E_m_). Additionally, recent work from our lab suggests that mouse, rat, and human *SLC9C1* possess conserved, and unique, methylation-sensitive DNA regulatory elements that contribute towards maintaining testis/sperm-specific expression [82].

Seminal work from the Garbers group [8] identified the first sperm-specific NHE and demonstrated its critical importance in mouse sperm motility and male fertility. The NHE10 (originally termed sNHE) cDNA was cloned from a mouse spermatid enriched cDNA library and the transcript was found to be exclusively expressed in testis in mouse and the NHE10 protein was found to localize to the principal piece of the mature mouse sperm flagellum [8].

### 2.2. Sperm Physiology and Fertility

NHE10 knockout (KO) mice exhibit normal testis and sperm morphologies but are completely infertile due to immotile sperm [8]. Interestingly, the cell permeable weak base NH_3_ is able to rescue motility in about 20% of the NHE10 KO mouse sperm and partially rescue their ability to fertilize zona pellucida-free eggs during in vitro fertilization (IVF) experiments, suggesting that the immotility phenotype is caused, at least partially, by an acidic intracellular pH [8]. However, there is ample evidence that lack of cAMP also underlies the loss of sperm motility and infertility in the NHE10 KO mouse. cAMP analogs rescue motility of NHE10 KO mouse sperm and partially restore their ability to fertilize zona pellucida-free eggs in IVF experiments [8]. NHE10 knockout sperm do not undergo normal protein tyrosine phosphorylation under capacitating conditions, but the addition of the cAMP analog Sp-cAMP along with the phosphodiesterase inhibitor 3-isobutyl-1-methylxanthine (IBMX) restore protein tyrosine phosphorylation levels back to the wildtype levels [80]. NHE10 KO mouse sperm have reduced basal levels of cAMP and HCO_3_^−^ sensitive cAMP synthesis is undetectable. Immunoblotting revealed that the full-length soluble adenylyl cyclase (sAC) protein is absent in NHE10 KO sperm, but levels of truncated sAC protein are similar in NHE10 KO and wild-type sperm. NHE10 appears to regulate the expression of full-length sAC at the protein level because the NHE10 KO and wild-type testes have comparable levels of full-length sAC mRNA. Cell culture experiments validated this hypothesis; co-transfection of NHE10 and sAC increased the expression of both the full length and truncated isoforms of sAC. It was also shown that expression of the carboxy terminus of NHE10 physically interacts with truncated sAC when coimmunoprecipitation studies were performed [80].

Optogenetics, using a transgenic mouse model that expresses a photoactivated adenylyl cyclase (bPAC) in the sperm, was used to further examine the functional relationship between sAC and NHE10 [83]. When bPAC mouse sperm are exposed to blue light, there is a rapid increase in intracellular cAMP synthesized by the transgenic photoactivated adenylyl cyclase. This bPAC mouse was bred with the NHE10 KO mouse background. When bPAC/NHE10 KO mouse sperm are exposed to blue light, there is a rapid increase in cAMP, sperm motility is restored, and the sperm are able to fertilize zona pellucida-intact oocytes in vitro [83]. All of this is evidence that in mice, NHE10 is not necessary for sperm motility and fertilization following an increase in cAMP via sAC. However, NHE10 is necessary for proper sAC expression and subsequent cAMP synthesis in mouse sperm. It is still unclear exactly what physiological role NHE10 has upstream of the increase in cAMP signaling events, although a recent study suggests that NHE10 expression is necessary for mediating the hyperpolarization-mediated alkalization observed in the mouse sperm flagellum [84]. Of note, addition of cAMP analogs is only able to partially rescue in vitro fertility, ~50%, and cAMP synthesis via pBAC is only able to rescue in vitro fertility ~30% [83]. This partial rescue of fertility in the absence of NHE10 protein suggests that NHE10 plays other roles in sperm to support motility and fertility.

### 2.3. NHE10 Transport Activity

Initial attempts to characterize the transport activity of the NHE10 protein failed due to the inability to express the NHE10 protein in cultured cells. Eventually, the Garbers group was able to show very weak expression of mouse NHE10 in a human cell line (HEK 293F) that was co-transfected with a truncated isoform of mouse sAC [80]. In an effort to increase NHE10 expression, the same group created a chimeric NHE1–NHE10 protein in which the first transmembrane domain of the mouse NHE10 protein was replaced with the first three transmembrane domains of the mouse NHE1 protein. When transfected into HEK293F cells, this chimeric NHE1–NHE10 protein was expressed and localized to the plasma membrane. Expression of the chimeric NHE1–NHE10 protein in a NHE1-null fibroblast cell line conferred resistance to acid-loading selection and the chimeric transporter was shown to possess weak Na^+^/H^+^ exchange activity [80]. However, since it is not clear whether the minimal NHE activity reported for the NHE1–NHE10 chimeric protein [80] is present without the contribution of the NHE1 components, characterization of the native mammalian NHE10 proteins is critical to understand how the transport activity of NHE10 influences mammalian sperm physiology.

Unfortunately, no unmodified mammalian NHE10 has been shown to possess NHE activity to date. However, sea urchins express a single SLC9C protein (originally identified as NHE10) that has recently been shown to be a bona fide NHE whose activity is not affected by the NHE inhibitors amiloride, EIPA, cariporide, and phloretin [81]. Mammals possess two SLC9C proteins (NHE10 and NHE11—see below) and the sea urchin protein is no more similar to mammalian NHE10 than mammalian NHE11 [43], making it impossible to determine whether it is a specific ortholog of either. Therefore, we will refer to the sea urchin isoform as the sea urchin SLC9C (suSLC9C) protein from here on.

Immunolocalization demonstrated that the suSLC9C protein resides in the plasma membrane of the entire sea urchin sperm flagellum as well as in part of the sperm head, potentially the acrosome [81]. The analyses of the suSLC9C protein also demonstrated that its NHE activity is enhanced in response to the egg peptide speract [81], confirming earlier work suggesting a voltage sensitive Na^+^/H^+^ exchange in sea urchin spermatozoa [85]. In addition, mass spectrometry revealed ~54,000 suSLC9C protein molecules in the flagellum of sea urchin *Arbacia punctulata* sperm, one of the most abundant proteins found in this study [86]. Such a concentration of suSLC9C protein molecules found in the flagellum would allow this sperm to rapidly fine tune pH_i_ in response to various cell signaling conditions.

The overall domain structure of the suSLC9C protein is predicted to be similar to the structure predicted for the mouse NHE10 in that they both contain an *N*-terminal NHE domain, followed by a VSD, and then a C-terminal CNBD; each domain containing many conserved functional residues [81]. Electrophysiological characterization of suSLC9C protein expressed in cultured cells revealed that this NHE is an electroneutral Na^+^/H^+^ exchanger that can exchange in either direction, depending on the ion gradient [81]. The VSD of the suSLC9C protein produces gating currents, suggesting that it is a functional voltage sensing domain. In addition, the NHE activity of the suSLC9C protein is voltage dependent with a V_1/2_ < −70.9 mV and cyclic nucleotides modulated the V_1/2_ of its activity: cAMP shifted the V_1/2_ of exchange activity to −56.8 mV and cGMP shifted the V_1/2_ of exchange activity to a lesser degree, to −67.8 mV [81]. These findings suggest that binding of cAMP to the CNBD of suSLC9C protein affects NHE activity by shifting the voltage dependence of activation. Although the suSLC9C protein has been confirmed to be a voltage and cyclic nucleotide-sensitive NHE, it was noted that specific amino acid residues in the suSLC9C protein VSD and CNBD, thought to be important for function, are not conserved in the mouse and human NHE10 orthologs [81]. Further studies are required to determine the activity of the mammalian proteins to determine whether mammalian NHE10 is an NHE that responds to changes in membrane potential and cyclic nucleotides to regulate sperm function similar to the suSLC9C protein in sea urchin sperm.

A recent study found that NHE10 KO mouse sperm are unable to alkalize their principal piece in response to valinomycin-induced hyperpolarization in the same manner that WT mouse sperm can [84]. These experiments suggest that NHE10 is responsible for the hyperpolarization-induced alkalization in mouse sperm and that the NHE10 protein is likely able to regulate pH and is sensitive to membrane potential. However, it was found that human sperm do not alkalize in response to valinomycin-induced hyperpolarization [84]. It is therefore possible that the human NHE10 activity is not regulated by membrane potential, but further characterization of the function of the VSD of human NHE10 is necessary.

### 2.4. NHE10 and Human Fertility

Although few specifics about the transport activity of mammalian NHE10 are known, evidence points to human NHE10 having a critical role in male fertility. A recent clinical study found significantly less NHE10 protein in sperm from asthenozoospermic compared to normozoospermic men and that NHE10 expression was positively correlated with higher sperm motility parameters [12]. It was noted in this study that NHE10 is specifically localized to the principal piece of the human sperm flagellum [12], similar to what is seen in mouse sperm [8]; however, human NHE10 is also reported to localize to the entire sperm flagellum [13]. Even more recent analysis of an infertile male presenting with asthenozoospermia found that this patient bares a homozygous mutation in *SLC9C1* [13]. The patient’s sperm displayed severely impaired motility as well as characteristics indicating membrane and flagellar fragility. Transmission electron microscopy (TEM) analysis revealed that the sperm exhibits midpiece defects but no acrosomal or axonemal structural abnormalities were observed. The specific mutation caused aberrant splicing around exon 22 which is predicted to cause an in-frame deletion of 33 amino acids in the consensus cyclic nucleotide binding domain of NHE10. The mutant NHE10 protein is still produced and was reported to localized, similar to the wild-type NHE10, to the entire length of the sperm flagellum [13]. It should be noted that this patient also bears mutations in two other genes and therefore the possibility that the infertile phenotype is due to a combination of these mutations cannot be excluded. In addition, since the level of sAC was not assessed in these mutant sperm, it is not clear whether the infertility of this patient is due to defective transport resulting from the mutant NHE10 protein, or if sAC protein expression is affected as is thought to occur in the mouse model completely lacking NHE10 [80]. While these findings highlight the importance of NHE10 in human male fertility, more work is needed to dissect the exact functions and interactions of human NHE10.

## 3. *SLC9C2* (NHE11)

### 3.1. Molecular Genetics and Expression Patterns

*SLC9C2* is located on human Chromosome 1 and is comprised of 28 exons. NHE11 exhibits a testis/sperm-specific expression pattern in both rats and humans [43]. Similar to mammalian NHE10, the rat and human NHE11 proteins are also predicted to contain seventeen total transmembrane domains, the first thirteen transmembrane domains encompassing a NHE domain and the final four transmembrane domains constituting a VSD. NHE11 protein expression is first detectable in spermiogenic cells and appears to colocalize with the developing acrosomal granule in both rat and human testis sections. Interestingly, NHE11 localizes specifically to the sperm head, likely the plasma membrane overlaying the acrosome, in both rat and human mature sperm cells. This is significant as no other NHE is known to localize to this region of the sperm head in mature sperm cells [43].

It is currently unclear what the evolutionary relationship is between *SLC9C1* and *SLC9C2*. Interestingly, in *Mus musculus*, *Slc9C2* is annotated as a pseudogene (designated *GM6185*).

### 3.2. Sperm Physiology and Fertility

No study to date has characterized the function of *SLC9C2* in any mammal as a *SLC9C2* knockout animal model is required.

### 3.3. NHE11 Transport Activity

No study to date has characterized the biochemical activity of the NHE11 protein. However, many residues previously demonstrated to be essential for Na^+^/H^+^ exchange in suSLC9C protein and human NHE1 are also conserved in mammalian NHE11, suggesting that mammalian NHE11 is likely a functional NHE. Additionally, many residues of the VSD and CNBD shown to be important for responses to changes in membrane potential and cyclic nucleotide binding are conserved in mammalian NHE11 [43]. All of this data suggests that mammalian NHE11 is likely a NHE regulated by both membrane potential and cyclic nucleotides, similar to sea urchin SLC9C protein. However, experiments are necessary to confirm this.

### 3.4. NHE11 and Human Fertility

Publicly available RNA-sequencing (https://www.ncbi.nlm.nih.gov/gene/284525, accessed on 10 January 2023 and https://orit.research.bcm.edu/MRGDv2, accessed on 10 January 2023) and proteomics data (https://www.proteomicsdb.org/protein/64851/expression, accessed on 11 January 2023) suggests that NHE11 is expressed exclusively in the testis and sperm in humans. Additionally, human NHE11 localizes to the head of mature sperm cells, the only known NHE to do so [43]. Finally, three separate studies that performed mass spectrometry on mature human sperm detected NHE11 [87,88,89]. Further studies are necessary to determine the physiological significance of human NHE11.

## 4. *SLC9B1* (NHA1/NHEDC1)

### 4.1. Molecular Genetics and Expression Patterns

*SLC9B1* is located on human Chromosome 4 and is comprised of 12 exons. *SLC9B1* appears to be the result of a tandem gene duplication event of an ancestral *SLC9B2* gene, appearing first in early marsupial mammalian evolution [90]. Interestingly, after this gene duplication event, *SLC9B1* gene expression evolved to exhibit testis/sperm-specific expression whereas *SLC9B2* displays an ubiquitous expression pattern [10,72,91]. Human *SLC9B1* is regulated by a single promoter with a CpG island that is methylation dependent [92]. This CpG island in the promoter region of *SLC9B1* is hypomethylated in human testis and hypermethylated in human lung tissue; suggesting that methylation of this CpG associated promoter region inhibits NHA1 expression in somatic cells [92].

NHA1 is exclusively expressed in testis in developing sperm in mouse, rat, monkey, goat, bull, and human [10,92]. NHA1 is reported to specifically localize to the principal piece in mouse sperm [10], but has also been reported to localize across the entire flagellum [11]. Cloning of mouse NHA1 cDNA and recombinant expression in cell culture showed that the NHA1 protein localizes to the plasma membrane [11].

### 4.2. Sperm Physiology and Fertility

NHA1 DNA vaccines given to female mice generated antisera and vaginal fluid containing proteins that specifically recognize the principal piece of wild-type mouse sperm and triggered sperm agglutination, acting as a crude contraceptive [10,91,93]. The role of NHA1 in male fertility has subsequently been supported and expanded by results of studies utilizing knockout mouse models.

The first of two lines of NHA1 knockout mice were generated using the Cre-LoxP system: mice with a NHA1 “floxed” allele were crossed with transgenic mice expressing Cre recombinase under the control of the zona pellucida 3 (Zp3) gene promoter. The Zp3 promoter drives the expression of Cre in the oocyte resulting in a global knockout of NHA1 from a conditional allele. These conditional NHA1 knockout (NHA1 cKO) mouse testes and sperm exhibit normal morphologies, and the sperm undergo the acrosome reaction at a rate comparable to wild-type sperm. However, these NHA1 cKO mice produced sperm with reduced motility resulting in subfertility [10]. The NHA1 cKO mouse testes and sperm also have diminished cAMP as well as reduced soluble adenylyl cyclase (sAC) protein levels, and the addition of cAMP analogs restores sperm motility to wildtype levels [10]. In transfected HEK293F cells, heterologous NHA1 expression increases endogenous sAC expression [10], reminiscent of what is seen in NHE10 transfected cell culture [80].

A second NHA1 knockout mouse model was generated and the knockout sperm display reduced motility and fertility [11], similar to the findings from the NHA1 cKO mice [10]. Interestingly, the sperm from this second NHA1 knockout were shown to have a stiff midpiece [11]. Analysis of this second NHA1 knockout model demonstrated that NHA1 activity, not NHE10, is involved in the zona pellucida-induced alkalization observed in capacitated sperm [11]. Specifically, when NHA1 knockout mouse sperm were exposed to soluble zona pellucida proteins, both pH_i_ and Ca^2+^ increases were strongly attenuated compared to the wildtype. Supporting the idea that NHA1 plays a role in pH_i_ and Ca^2+^ regulation in mouse sperm, previous studies demonstrated that wild-type mouse sperm treated with anti-NHA1 antisera displayed decreased motility as well as reduced pH_i_ and intracellular Ca^2+^ concentration [91]. However, db-cAMP (a cell permeant, non-hydrolyzable cAMP analog) did not rescue the pH_i_ and Ca^2+^ response to zona pellucida protein exposure in the second line of NHA1 knockout mice [11]. This suggests that NHA1 function is either directly or indirectly responsible for both pH_i_ and Ca^2+^ regulation in response to zona pellucida protein stimulation. Furthermore, wildtype and NHA1 knockout sperm beat frequency (with and without HCO_3_^−^) was similar [11]. These findings suggest that loss of NHA1 activity/expression underlies the abnormal phenotype of the NHA1-deficient sperm, rather than defective sAC functionality leading to impaired cAMP synthesis. Interesting, residual NHE activity (measured as intracellular alkalization) in response to zona pellucida proteins in NHA1 knockout sperm was observed and was suggested to be a contribution of either an unknown NHE, NHA2, or some combination of both [11]. This may explain why NHA1-deficient male mice are not completely infertile; there may be other NHE(s) that share functional redundancy with NHA1.

Two other vital ion transporters in mammalian sperm are the CatSper complex and the Slo3-Lrrc52 complexes. The CatSper complex forms a channel that is responsible for Ca^2+^ influx into the cell that is required for capacitation. CatSper current is activated by an increase in pH_i_, E_m_, and progesterone in humans [31,94] but only by pH_i_ and E_m_ in mice [30]. The Slo3-Lrrc52 complex is responsible for the K^+^ influx observed in capacitation that in turn hyperpolarizes the E_m_ in mouse and human sperm, ultimately regulating pH_i_ and Ca^2+^ influx [25,26,95,96,97,98,99]. CatSper organization and density is similar in wildtype and NHA1 knockout sperm as measured by CatSper1 immunofluorescence staining, suggesting that the reduced motility and fertility is not due to deficient CatSper-mediated signaling [11]. Intriguingly, using Slo3 knockout or Lrrc52 knockout mice, both of which exhibit improperly depolarized E_m_ sperm phenotypes, it was shown that zona pellucida induced pH_i_ and Ca^2+^ responses were abolished [11]. Since Slo3 and Lrrc52 function together to establish a hyperpolarized membrane potential in capacitated sperm [95,96], this finding suggests that the pH_i_ and Ca^2+^ responses to zona pellucida proteins mediated by NHA1 requires a sufficiently negative E_m_ [11]. Zona pellucida proteins do not evoke an increase in cAMP synthesis, however cAMP synthesis by sAC is required upstream to prime NHA1 for activation in response to zona pellucida proteins [11]. How NHA1 is activated by zona pellucida proteins, E_m_, or cAMP remains unknown, and it is unclear if NHA1 plays a role in sperm physiology outside of zona pellucida induced alkalization.

### 4.3. NHA1 Transport Activity

To date, no mammalian NHA1 protein has been characterized with respect to transport activity, however the *Drosophila melanogaster* NHA1 protein is known to act as a Cl^−^/H^+^ exchanger when expressed in *Xenopus* oocytes [100]. The *Drosophila melanogaster* NHA1 protein has been shown to be widely expressed across multiple tissues and its sequence is more similar to human NHA2 than human NHA1. *Drosophila* NHA1 activity is sensitive to the anion exchange inhibitors 4,4′-dibenzamido-2,2′-stilbenedisulphonate (DBDS) and 4,4′-diiso-thiocyano-2,2′-disulfonic acid stilbene (DIDS), but not to the NHE/Na^+^ channel inhibitor amiloride [100]. On the other hand, the *Drosophila* NHA2 protein sequence is more similar to that of the human NHA1 protein and *Drosophila* NHA2 was shown to be a functional NHE [100]. Knockdown of both NHA1 and NHA2 in *Drosophila* was lethal due to salt intolerance [100]. A recent study also characterized the activity of NHA1 from the red flour beetle, *Tribolium castaneum*, and found that this protein functions as an electroneutral H^+^/K^+^ antiporter [101]. However, *Tribolium castaneum* only possess one *SLC9B* homolog, and similar to *Drosophila* NHA1, the *Tribolium* NHA1 protein shares more sequence homology with mammalian NHA2 than NHA1. Therefore, direct comparison of the insect NHA proteins to mammalian NHA1 or NHA2 proteins should be undertaken with care and further research into directly characterizing the transport activity of mammalian NHA1 is of importance. However, the finding that NHA1 KO mice exhibit defects in pH_i_ regulation in response to zona pellucida stimulation strongly suggests that at least mouse NHA1 exhibits NHE activity (assuming that loss of NHA1 expression does not also result in loss of other pH regulating proteins) [11].

A structural model of human NHA1 was generated based on homology to bison NHA2 [73,77]. This human NHA1 model was overall identical to bison NHA2, and the structure of the ion-binding sites were identical. Importantly, the bison NHA2 has been shown to be a functional NHE (see NHA2 below) and all of the residues within the binding site with assigned roles for Na^+^ and H^+^ binding are also conserved in human NHA1. However, some residues proposed to function in phosphatidylinositol (PI) or phosphatidylinositol diphosphate (PIP_2_) binding in bison NHA2 are not conserved in human NHA1. Additionally, human NHA1 possesses different residues than those in bison NHA2 that are thought to be involved in the cytoplasmic dimerization interface. All of these data suggest that human NHA1 and bison NHA2 likely possess similar ion transport (NHE) activity, but may be regulated differently through their interaction with lipids and the stability of the dimer interface itself [73].

### 4.4. NHA1 and Human Fertility

NHA1 has been shown to be expressed in human testis via Western blotting and immunohistochemistry [92]. Additionally, immunofluorescence revealed that NHA1 localizes to the principal piece in human sperm, the same localization as seen in mouse sperm [10]. NHA1 has also been reported to be present in the human sperm proteome [87,89]. Finally, a bioinformatics study examining recent population-specific positive directional selection of human male reproductive genes found that *SLC9B1* is under positive directional selection in all studied human populations. [102]. Taken together, these findings suggests that NHA1 is likely important for human male fertility, however, more work is needed to elucidate the specific role of this NHE in human sperm.

## 5. *SLC9B2* (NHA2/NHEDC2)

### 5.1. Molecular Genetics and Expression Patterns

*SLC9B2* is located in tandem with *SLC9B1* on human Chromosome 4 and is comprised of 13 exons. In contrast to *SLC9B1*, *SLC9B2* exhibits near ubiquitous expression, including expression in testis and sperm, in both mouse and human [6,10,74,75]. In mature mouse sperm, NHA2 has been reported to reside in the principal piece [10].

NHA2 subcellular location seems to be widespread as it has been reported to localize to multiple cellular compartments in various cells. In mouse pancreatic β-cells, NHA2 localizes to endosomes and synaptic-like microvesicles and, similarly, was found to localize to late endosomes and lysosomes in mouse osteoclasts [103,104]. NHA2 is also reported to localize to mitochondria in both mouse osteoclasts and rat kidney cells [74,105]. Additionally, NHA2 was reported to localize to the plasma membrane of mouse kidney cells, mouse osteoclasts, and MDCK cells (canine kidney cells) [75,76,104,106].

Physiologically, NHA2 has been shown to be important for osteoclast function, insulin secretion, and nephron function where it has been implicated in playing a significant role in maintaining salt balance to support normotension [75,103,104,107]. NHA2 was reported to localize to the distal convoluted tubules of mouse kidney cells, and subcellularly, was found to localize to endosomes in mpkDCT4 cells, a mouse distal convoluted tubule epithelial cell line, although NHA2 was found to not influence endosomal pH homeostasis [107]. NHA2 knockout mice display a Gitelman syndrome-like phenotype, characterized by reduced blood pressure, normocalcemic hypocalciuria, and increased urinary aldosterone excretion. Mechanistically, NHA2 appears to regulate blood pressure through a WNK4-NCC pathway in the mouse kidney [107]. For a review on NHA2 work from the Reymond, von Ballmoos, and Fuster groups, see [108].

### 5.2. Sperm Physiology and Fertility

As with NHA1, two lines of NHA2 KO mice have been generated. The Liu group generated conditional NHA2 knockout mice in the same manner as the NHA1 conditional knockout mice [10] such that a conditional global knockout of NHA2 occurred. The NHA2 conditional knockout (NHA2 cKO) mice exhibited a very similar phenotype to the NHA1 cKO mice in that they were subfertile due to reduced sperm motility. Similar to the NHA1 cKO mice, there are no grossly abnormal testis or sperm morphologies and the sperm have reduced cAMP levels and sAC protein levels. Unfortunately, there is no information available regarding whether cAMP analogs can restore motility and fertility of NHA2 cKO sperm. Of interest, the NHA1 cKO mouse testis had significantly upregulated NHA2 mRNA and the NHA2 cKO mouse testis had significantly upregulated NHA1 mRNA, suggesting a functional redundancy for the two transporters [10]. Interestingly, there are no reports of a subfertile male phenotype from the second group that generated NHA2 knockout mice [103,107].

NHA1/NHA2 double knockout mice (NHA1/2 dKO) were reported to have been generated [10] however, as noted by [73] the likelihood of generating these NHA1/2 dKO by cross-breeding the NHA1 cKO and NHA2 cKO mice is low because *Slc9B1* and *Slc9B2* are located in tandem on chromosome 3 in mice. These two genes are separated by less than 18,500 bp on mouse chromosome 3 (according to the chromosomal locations for these two genes provided in [90]), making the occurrence of a crossover event happening that would result in positioning both of the individual knockout alleles on the same chromosome extremely rare. Therefore, it would be expected to take a cumbersomely large number of matings between the single KO mice to generate the NHA1/2 dKO founder and thus the actual genotypes of the NHA1/2 dKO animals used in these studies are unclear. In any case, the NHA1/2 dKO sperm were reported to have almost no motility resulting in completely infertile males. Likely because the NHA1/2 dKO mouse sperm were reported to have significantly reduced cAMP and sAC protein levels [10]. Similar to the NHA2 cKO sperm, there is no information about the ability of cAMP analogs to restore motility to the NHA1/2 dKO sperm.

### 5.3. NHA2 Transport Activity

Initial attempts to characterize the transport activity of the human NHA2 protein found that heterologous expressed NHA2 in salt-sensitive yeast strains was able to transport either Na^+^ or Li^+^, consistent with conventional Na^+^/H^+^ exchanger activity [74,109]. Later characterization of human NHA2 was accomplished by overexpressing green fluorescent protein (GFP)-tagged human NHA2 fusion protein in MDCK cells and it was found that NHA2 acts to export Li^+^ out of the cell in exchange for the import of Na^+^, however, in the presence of a high extracellular proton concentration (acidic conditions outside the cell) NHA2 could mediate Li^+^ efflux in exchange for H^+^ import. This NHA2 activity was inhibited by cytosolic acidification as no Na^+^ influx was seen after the cells were acid loaded [76]. In addition, there appears to be a consensus that NHA2 does not transport K^+^ and that its activity is not electrogenic [74,76,78]. Furthermore, NHA2 activity is resistant to amiloride but sensitive to the sodium–glucose transporter inhibitor phloretin [75,76]. Interestingly, exogenously expressed NHA2 coimmunoprecipitated with the V-type H^+^-ATPase in a kidney cell line and the V-type H^+^-ATPases inhibitor bafilomycin was able to block NHA2-mediated Li^+^ tolerance [76]. These findings led to a model proposing that NHA2 cation extrusion is driven by the proton gradient setup by the H^+^ efflux driven by the V-type H^+^-ATPase [76], therefore suggesting that human NHA2 is a H^+^-driven cation (Na^+^/Li^+^) exporter with activity similar to the prokaryotic NHAs. It is currently unclear whether NHA2 and V-Type H^+^-ATPases are also functionally coupled in spermatozoa; future experiments are needed to examine whether these proteins share similar transport characteristics in sperm and kidney cells.

More recent experiments found that human NHA2 preferentially forms stable homodimers in membranes [79] and exhibits electroneutral NHE activity when in proteoliposomes [78]. In addition, the structure of the bison NHA2 protein was recently solved and found to possess 14 transmembrane domains and form a homodimer [77]. Solid-state membrane-based electrophysiology experiments on proteoliposome-reconstituted bison NHA2 revealed that bison NHA2 mediates Na^+^/H^+^ exchange (can also transport Li^+^/H^+^) but in a pH dependent manner, specifically Na^+^(Li^+^)/H^+^ exchange was severely inhibited by a low pH outside the liposome relative to inside the liposomes. All together, these findings suggest that NHA2 operates as an electroneutral exchanger to drive Na^+^ efflux [77]. However, biochemical analyses of NHA2 in its native environment is lacking and whether or not NHA2 plays a role in organellar pH homeostasis such as endosomal pH regulation needs to be clarified. Additionally, how NHA2 transport activity influences sperm physiology remains unknown.

### 5.4. NHA2 and Human Fertility

A recent study detected NHA2 protein in human sperm via mass spectroscopy [89], however its exact contribution to human male fertility is still unknown.

## 6. *SLC9A1* (NHE1)

### 6.1. Molecular Genetics and Expression Patterns

*SLC9A1* is located on human Chromosome 1 and is comprised of 12 exons [110]. Due in part to its near ubiquitous expression pattern, NHE1 is often referred to as the “housekeeping” NHE involved in pH homeostasis and cell tonicity [6]. NHE1 has been shown to localize to various subregions of the plasma membrane in different cell types and in response to various stimuli. For example, NHE1 localizes to caveolae and other cholesterol enriched microdomains, such as lipid rafts [6,111,112].

### 6.2. Sperm Physiology and Fertility

NHE1 localizes to the midpiece in mature rat sperm [51] and has also been shown to localize to both the equatorial region of the sperm head and to the sperm flagellum in ram and pig sperm [113,114]. NHE1 KO mice exhibit ataxia, growth retardation and seizures evident around 2 weeks of age [7]. Due to repeated seizures, many NHE1 KO mice die before reaching sexual maturity, limiting the investigation of NHE1’s role in sperm physiology. Despite these limitations, NHE1 KO male mice that do reach sexual maturity are capable of siring offspring when mated with wild type female mice [7], which suggests that NHE1 is not critical for sperm physiology as complete loss of NHE1 in males does not alter murine fertility.

### 6.3. NHE1 Transport Activity

The mature NHE1 protein localizes to the plasma membrane and preferentially homodimerizes but only one functional copy of the dimer is needed for effective Na^+^/H^+^ exchange at an acidic pH [115,116]. The human NHE1 protein structure was recently solved by co-expressing NHE1 and the obligate binding partner calcineurin B-homologous protein 1 (CHP1) in HEK293 cells, and then reconstituting the NHE1-CHP1 complex in lipid nandodiscs and determining its structure by cryoelectron microscopy (cryo-EM) [50]. Human NHE1 was found to possess 13 transmembrane domains [50]. The intracellular C-terminus of the NHE1 protein acts as a pH sensor to allow for appropriate regulation of NHE activity, possibly through dimeric interaction between the C-termini of interacting NHE1 molecules [117,118]. NHE1 transport activity has been extensively characterized and it has been shown to exhibit either 1:1 or 2:2 stoichiometric exchange of extracellular Na^+^ for intracellular H^+^ [58,119] and is able to exchange in reverse mode if the ion gradients are reversed [120]. Of potential interest in the context of sperm physiology due to the importance of Ca^2+^ during capacitation, a human NHE1–Calmodulin (CaM) complex was solved by nuclear magnetic resonance (NMR) spectroscopy and it was found that NHE1 transport activity was regulated both by CaM and intracellular Ca^2+^ concentrations [121]. However, it should be noted that NHE1 has been found to be regulated by CaM differently in different cell-specific and NHE1 phosphorylation state-specific manners [121,122,123,124], and therefore how or if NHE1 activity is regulated by Ca^2+^, CaM binding, or phosphorylation in mammalian sperm is currently unknown but is a relevant future area of research.

NHE1 activity is highly sensitive to amiloride, ethylisopropoylamiloride (EIPA), 5-(*N*,*N*-dimethyl)-amiloride (DMA), and 5-(*N*,*N*-hexamethylene)-amiloride (HMA), and cariporide, all known inhibitors of NHEs (for reviews of NHE inhibitor structures, specificity and applications see) [6,7,59,60,62,125,126]. For a detailed review on NHE1 structure and transport see [61].

### 6.4. NHE1 and Human Fertility

Mass spectroscopy analysis revealed that NHE1 is present in the mature human sperm proteome [127], however, no studies on the contribution of human NHE1 to male fertility exist. Several clinical studies have characterized homozygous mutations in human *SLC9A1* which result in loss of function of the NHE1 protein due to improper protein transport/exchange [128,129,130]. These patients suffer from ataxia or other central nervous system (CNS) deficiencies [128,129,130], however fertility phenotypes associated with loss of human NHE1 activity have not been reported.

## 7. *SLC9A3* (NHE3)

### 7.1. Molecular Genetics and Expression Patterns

*SLC9A3* is located on human chromosome 5 and is comprised of 17 exons. The NHE3 protein is most highly expressed in the digestive tract and kidney with expression in other tissues, including testis. NHE3 is known to undergo regulated insertion and retrieval between the plasma membrane and recycling endosomes, depending on the cellular context [6]. It is believed that NHE3 is either directly or indirectly responsible for the majority of the absorption of ingested Na^+^ in the small and large intestines as well as reabsorbing over half of the filtered Na^+^ in the proximal tubules of the kidneys [63]. For a detailed review on the role of NHE3 in the digestive tract and kidney, see [63].

### 7.2. Sperm Physiology and Fertility

NHE3 was first reported to be expressed in the male reproductive tract in the efferent duct and proximal regions of the epididymis in rat [131] and the efferent duct in human [132] and mice [64]. NHE3 KO male mice are infertile due to an abnormally dilated lumen of the rete testis and efferent tubules resulting in obstructive azoospermia [15,64]. Further analyses revealed that this phenotype is likely due to the significant decrease in transmembrane conductance regulator (CFTR) protein expression in the epididymis and vas deferens of NHE3 KO mice [65]. NHE3 and CFTR are known to interact in various cellular contexts [133,134], and therefore it is thought that the NHE3 KO phenotype is at least partially due to the loss of CFTR activity [65], as CFTR KO mice also display a similar male infertility phenotype [135,136].

NHE3 has also been shown to be expressed in spermiogenic cells in mouse testis, and found to specifically localize to the developing acrosomal granule [42]. Interestingly, NHE3 KO male mice produce sperm with severe acrosome defects, suggesting that NHE3 protein expression is essential for proper acrosome biogenesis. It should be noted that NHE3 has not been reported to be expressed in mature murine sperm. All of this data on NHE3 KO mice makes it clear that NHE3 expression is important for male fertility, both due to its role in acrosome biogenesis and its role in maintaining proper luminal fluid homeostasis in the epididymis and vas deferens [15,42,64,65]. However, whether NHE3 plays a functional role in mature sperm has yet to be reported.

### 7.3. NHE3 Transport Activity

The transport activity of the NHE3 protein has been extensively characterized via heterologous expression of NHE3 cDNA in cell culture systems. Rat NHE3 cDNA expressed recombinantly in an NHE-deficient cell line was found to function as an electroneutral NHE. When comparing rat NHE1 with rat NHE3 activity, it was found that NHE3 exhibits a higher affinity for Na^+^ than NHE1 but a lower affinity for H^+^. Additionally, NHE3 is significantly less sensitive to amiloride inhibition than NHE1 [62,66]. Potentially relevant to sperm physiology, NHE3 transport is decreased in response to increases in intracellular cAMP concentrations; cAMP activates protein kinase A (PKA) which leads to phosphorylation of serine residues within the cytoplasmic C-terminus of NHE3 to inhibit its activity [137,138,139]. How and if NHE3 is also inhibited via cAMP in testis or sperm is unknown, but would be functionally relevant. For a detailed review on regulatory binding partners and complexes of NHE3, see [140].

The structure of the human NHE3 protein was recently solved by co-expressing human NHE3 with human CHP1 and then the NHE3–CHP1 complex was reconstituted into nanodiscs and resolved with cryo-EM (in a similar manner to how the NHE1–CHP1 complex was solved) [67]. Results of this study show that NHE3 assembles as a homodimer, with each NHE3 monomer containing 13 transmembrane domains. This study also found that CHP1 co-expression enhances NHE3 trafficking to the plasma membrane and subsequently increased its transport activity. Additionally, NHE3 possesses an autoinhibitory domain, referred to as a plug motif, formed from a cytosolic helix-loop-helix motif that is thought to compete with substrate binding to inhibit transport. This autoinhibitory domain of NHE3 is apparently modulated by binding of auxiliary proteins or by phosphorylation to prevent or destabilize this autoinhibition, allowing for fine-tuning of NHE3 transport activity under specific cellular conditions [67].

### 7.4. NHE3 and Human Fertility

Human males with *SLC9A3* mutations resulting in loss of NHE3 expression exhibit a congenital bilateral absence of the vas deferens (CBAVD) phenotype, reminiscent of the phenotype of the NHE3 KO mice [14,65]. Whether or not NHE3 is also expressed in human testis and sperm, specifically the developing acrosome, has yet to be reported and therefore NHE3’s role in human spermatogenesis is unclear. However, the CBAVD phenotype observed in human males with *SLC9A3* mutations demonstrates the importance of this NHE for proper male fertility due to its posited role in maintaining proper fluid homeostasis in the vas deferens.

## 8. *SLC9A5* (NHE5)

### 8.1. Molecular Genetics and Expression Patterns

*SLC9A5* is located on human Chromosome 16 and is comprised of 16 exons. The NHE5 protein is predicted to be most similar to the NHE3 protein by sequence analysis and similar to NHE3, has been shown to localize to both the plasma membrane and recycling endosomes [6,141]. Analysis of mouse, rat, and human tissue RNA expression revealed NHE5 is most highly expressed in the brain, with second highest expression found in the testis and low levels expressed in various other tissues such as spleen and lung [6,68,142].

### 8.2. Sperm Physiology and Fertility

NHE5, along with NHE1, is localized to the midpiece of the sperm flagellum in rats [51]. An NHE5 knockout mouse model was generated and these mice display increases in memory and learning [69]. While the interest of this study was not the of the role of NHE5 in sperm, the authors did report that the NHE5 KO mice displayed no fertility issues, suggesting that NHE5 may not be an essential protein for male fertility in mice. Future studies using these NHE5 knockout mice as a model to investigate specific aspects of male fertility such as motility, capacitation, and fertilization would be of great interest to further elucidate the role of NHE5 in mammalian sperm. Further, since NHE1 and NHE5 both localize to the midpiece of the sperm and therefore could compensate for each other, studies of NHE1/NHE5 double knockout mice may be needed to uncover the role of these two NHE subfamily members in these cells.

### 8.3. NHE5 Transport Activity

NHE5 cDNAs cloned from rat and human brain expressed in NHE-deficient cells were used to characterize NHE5 transport activity. Both rat and human NHE5 exhibit similar transport kinetics to NHE3 in that NHE5 is able to regulate pH_i_ by exchanging extracellular Na^+^ for intracellular H^+^, likely in an electroneutral manner [142,143]. Interestingly, rat NHE5 is inhibited by acute exposure to the cell permeant, non-hydrolyzable cAMP analog 8-bromo-cAMP or to forskolin (which stimulates adenylate cyclase to synthesize cAMP), while the PKA inhibitor H-89 is able to prevent this cAMP-mediated NHE5 inhibition. The PKC activator phorbol 12-myristrate 13-acetate inhibits rat NHE5 while the PKC antagonist chelerythrine chloride blunts this effect [143]. Taken together, these findings suggest that both PKA and PKC act to inhibit rat NHE5 activity, although the mechanism behind this inhibition is currently unknown. A study using both neuronal and non-neuronal cells found that activated AMP-activated Protein Kinase (AMPK) phosphorylates human NHE5 which causes it to be recruited to the plasma membrane to regulate pH_i_ under specific conditions such as metabolic stress [144]. AMPK localizes to different parts of mature sperm in mouse, bull, and human, one location being the midpiece [145] where NHE5 is present in mice and rats. AMPK plays a role in energy homeostasis in mammalian sperm which directly contributes to sperm motility and thus fertility [145]. Additional studies are needed to determine whether NHE5 is regulated by AMPK in the midpiece of mammalian sperm, if so, this could suggest a role for NHE5 in sperm energy homeostasis.

Both rat and human NHE5 activities are relatively insensitive to amiloride and slightly sensitive to EIPA, similar to the sensitivities of NHE3 to these NHE inhibitors [68,142,143]. Using these pharmacological inhibitors to understand the role of NHE5 activity in mammalian sperm is not possible because NHE5 is less sensitive to inhibition by them than NHE1. Therefore, the higher inhibitor concentrations needed to block NHE5 activity will also inhibit NHE1 (as well as other potential Na^+^ transporters in sperm) and therefore any observed differences could not be attributed specifically to loss of NHE5 activity. It should be noted however, that incubating rat sperm with amiloride inhibits motility in a concentration-dependent manner (with an IC50 of 5 × 10^−5^ M) [2] suggesting that a relatively amiloride-resistant NHE (such as NHE5) [59] may be involved.

### 8.4. NHE5 and Human Fertility

Nothing has been reported regarding NHE5 and human male reproduction other than a mass spectroscopy analysis which revealed the presence of NHE5 in mature human sperm [146]. Therefore, more work is needed to confirm NHE5’s presence in human sperm and to analyze its contribution to male fertility.

## 9. *SLC9A8* (NHE8)

### 9.1. Molecular Genetics and Expression Patterns

*SLC9A8* is located on human Chromosome 20 and is comprised of 16 exons. The NHE8 protein is ubiquitously expressed and in somatic cells has been shown to localize to the trans-Golgi network and to function to regulate late endosomes by maintaining intra-Golgi pH [6,16,60,147]. In normal rat kidney (NRK) cells, the surface expression of NHE8 increases in response to acidic media via increased trafficking to the plasma membrane [148]. Thus, NHE8 displays roles in both the plasma membrane and intracellular compartments [6,44]. For a detailed review on NHE8, see [60].

### 9.2. Sperm Physiology and Fertility

NHE8 mRNA and protein have been reported to be expressed in both mouse and human testis and NHE8 protein was shown to be expressed in mouse and human Leydig cells and was shown to localize intracellularly in MLTC-1 cells (a mouse Leydig cell line) [71].

Two separate groups have independently generated NHE8 knockout mice and both groups reported that male NHE8 knockout mice were completely infertile [9,71,149]. The first of these knockout mouse studies found that global knockout of NHE8 resulted in loss of sperm production due to impaired Leydig cell function [71]. The second NHE8 knockout mouse study found that both global knockout and germ-line specific knockout of NHE8 resulted in identical phenotypes; the NHE8 knockout sperm were round-headed, lacked acrosomes, and had incorrectly localized mitochondrial sheaths [9]. Sertoli cell specific NHE8 knockout resulted in mice with morphologically normal sperm that were fertile [9]. Importantly, this study also reported that NHE8 colocalizes with the acrosome during all stages of spermiogenesis in wild-type mouse [9]. Although both NHE8 knockout mouse studies resulted in infertile males, there seems to be some discrepancies between these studies. The Oberheide group appears to clearly show that NHE8 loss-mediated infertility comes from a germ cell specific origin and that NHE8 expression is present in developing wild-type mouse spermatozoa, which the Xu group does not report. Additionally, the Oberheide group reports an absence of any NHE8 expression in Leydig cells, thus the role of NHE8 in these cells is still in question. Regardless of the discrepancies, both NHE8 knockout studies demonstrate that NHE8 is a vital protein to male fertility. Future research is needed to determine what the exact role of NHE8 is in acrosome formation during spermiogenesis and if NHE8 has a role in mature sperm as its expression in mature sperm has not been reported.

### 9.3. NHE8 Transport Activity

Initial cloning and expression of human NHE8 and subsequent reconstitution into proteoliposomes showed that NHE8 does possess Na^+^/H^+^ exchange activity [16]. Additionally, NHE8 was found to function as a NHE that is sensitive to EIPA however these experiments used EIPA concentrations that would also minimally inhibit NHE1 activity in AP-1 cells [70]. A recent study characterized the transport activity of both rat and human NHE8 in the NHE-deficient PS120 cell line, and found both rat and human NHE8 function as NHEs that are highly sensitive to the NHE1 inhibitor cariporide (HOE642) [150].

### 9.4. NHE8 and Human Fertility

Immunohistochemical labeling of NHE8 in the Leydig cells of human testis has been reported [71]. However, there is currently no data on the expression of NHE8 in human sperm or of its contribution to human male fertility. The phenotype of NHE8 knockout mouse sperm (rounded heads that lack acrosomes) is a very similar to the clinical condition of globozoospermia that affects some human males, suggesting that NHE8 may be a genetic candidate for clinical globozoospermia diagnosis [9].

## 10. Discussion

### 10.1. NHE10/11’s Likely Functional Dependence with SLO1/3-Mediated Hyperpolarization in Mammalian Sperm

Membrane hyperpolarization, as part of capacitation events, is of demonstrated importance for the fertilizing ability of both human and mouse sperm [25,28,29,95,151,152]. Human sperm that exhibit depolarized membrane potentials have low fertilization rates during IVF [153,154] and hyperpolarization after capacitation is a strong predictor for the acrosome reaction, hyperactivity, and successful IVF [28,29]. Using fluorimetry to measure human sperm membrane potential demonstrated that non-capacitating sperm have an average E_m_ of −37.7 ± 9.9 mV, while capacitated sperm have an E_m_ of −57.8 ± 12.9 mV [29]. Similarly, flow cytometry revealed that non-capacitated human sperm have an E_m_ of −35.7 ± 2.8 mV and capacitated sperm have an E_m_ of −45.2 ± 3.2 mV. Potentially more physiologically important, fertile human sperm have an E_m_ of −58.03 ± 3.00 mV while non-fertile sperm have an E_m_ of −40.61 ± 2.60 mV [28]. Although little is known about the activity of the mammalian SLC9C proteins, these E_m_ values of capacitated/fertile human sperm are consistent with values at which the suSLC9C protein has been shown to become active in the absence of cNMP (V_1/2_ of −70.9 ± 2.5 mV) and in the presence of cAMP (V_1/2_ of −56.8 ± 2.7 mV) [81]. This shift to a more depolarized activation of suSLC9C in the presence of cAMP suggests that the suSLC9C protein may only be active post capacitation, when cAMP levels rise due to sAC activity and the E_m_ hyperpolarizes. The transport activity of the mammalian SLC9C proteins (NHE10 and NHE11) have yet to be determined, however, if they share similar voltage and cyclic nucleotide regulation as their sea urchin orthologue, the NHE10/11 proteins could be responsible for pH regulation in mammalian sperm that have undergone membrane hyperpolarization in response to capacitating conditions.

Studies using Slo3 and NHE10 knockout animals support the idea that NHE10 regulates sperm pH_i_ in response to membrane hyperpolarization in mice. First, it appears that Slo3 K^+^ channels regulate Ca^2+^ entry via CatSper in mouse sperm through membrane hyperpolarization by regulating sperm K^+^ current (KSper); Slo3 KO mouse sperm do not display CatSper-mediated Ca^2+^ currents unless external pH is increased or the E_m_ is artificially hyperpolarized with an ionophore [25]. In addition, treatment of wild-type mouse sperm with valinomycin, a K^+^ selective ionophore which causes artificial hyperpolarization of the sperm E_m_, induces a pH_i_ increase, further supporting the idea that mouse sperm pH_i_ is regulated by membrane voltage change and leading to the suggestion that a voltage dependent NHE (NHE10) may be responsible for activating CatSper in response to the hyperpolarization evoked from Slo3 channels opening [25]. Additional experiments found that when the inward Na^+^ gradient was depleted, mouse sperm cells still undergo hyperpolarization but the CatSper mediated Ca^2+^ current is absent, further evidence supporting possible NHE activity in the activation of CatSper [25]. A recent study found that WT mouse sperm undergo cytosolic alkalization in the sperm flagellum under valinomycin-induced E_m_ hyperpolarization, but NHE10 KO mice do not [84], demonstrating that in mouse, NHE10 expression is required for the hyperpolarization-induced alkalization. Interestingly, simply incubating mouse sperm in alkaline conditions is able to activate Slo3 mediated hyperpolarization [155], suggesting that the journey through the female reproductive tract, in which the sperm encounter a progressively increasing external pH, may be enough to influence Slo3 channel activation and subsequent E_m_ hyperpolarization.

While human sperm also require membrane hyperpolarization to activate CatSper-mediated Ca^2+^ entry, unlike mouse sperm, they do not undergo cytosolic alkalization in response to valinomycin-induced hyperpolarization [84]. Another study that performed kinetic patch-clamp fluorometry on human sperm found that, unlike sea urchin sperm, these human sperm cells did not alkalize in response to either hyperpolarization or cAMP [89]. This data suggests that humans may have evolved a different regulation of their SLC9C proteins in sperm. In addition, the K^+^ channel responsible for Ca^2+^-mediated human sperm hyperpolarization is still not definitively identified [156]. It has been suggested that this channel could be SLO1 because SLO1 channels are Ca^2+^ sensitive while SLO3 are generally not [27]. It has also been suggested that SLO3 is responsible for the observed human sperm hyperpolarization because unlike mouse Slo3, human SLO3 is only weakly activated by an increase in pH_i_, but strongly activated by an increase in Ca^2+^ from CatSper [26]. Both SLO1 and SLO3 are reported to be present in human sperm [26,27], and therefore it is unclear what each ion channel’s role is in sperm hyperpolarization, even though it is clear that K^+^ efflux (mediating membrane hyperpolarization) is important for human sperm capacitation and fertilization [99,152,157]. Of note, a recent study that performed in depth mass spectroscopy analysis of the human sperm proteome failed to detect SLO1 [89].

### 10.2. Interaction of NHEs and sAC in Sperm

Cell-permeant, cAMP analogs restore motility and fertility to NHE10 KO sperm [80], NHE10 and sAC co-immunoprecipitate from mouse and sea urchin sperm [80,158], and in cultured cells, the heterologous expression of mouse sAC and NHE10 are mutually dependent [80] suggesting that these two proteins are functionally linked. Interestingly, in sea urchin sperm, sAC co-immunoprecipitates with the suSLC9C protein along with other known plasma membrane and axonemal proteins [158]. These findings suggest that in the sea urchin sperm flagellum, suSLC9C and sAC, along with multiple other proteins, form a large protein complex that would allow an increase in cAMP generated by sAC to efficiently shift the activation voltage of this NHE to a more positive, physiologically relevant V_m_ via interaction with the CNBD of the suSLC9C protein located in the same complex [81]. Mammalian NHE10 and NHE11 both are predicted to possess CNBDs with conserved residues important for cyclic nucleotide binding [8,43], and therefore are also equipped to be regulated by the increase in cAMP mediated by sAC stimulation in capacitation events.

### 10.3. Functional Interdependence of CatSper and NHEs

As mentioned, the CatSper complex is responsible for the Ca^2+^ influx into the cell that is required for capacitation. Alkalization shifts the activation threshold of CatSper to more negative physiologically relevant potentials [30,31,94]. In mouse sperm, CatSper is activated by increases of pH_i_ and is E_m_ sensitive [30,34]. In human sperm, CatSper current is also E_m_ sensitive and is strongly activated by alkalization, however unlike in mouse sperm, progesterone enhances CatSper current without directly influencing pH_i_ [31,94,159]. The dependence of CatSper activation on pH_i_ increase strongly implicates the activity of pH regulating proteins, such as NHEs, in this process.

Interestingly, a comparative genomic study found that nearly all animals that possess at least four α pore-forming subunit CatSper genes also have both sAC and at least one *SLC9C* gene [47]. Species that do not have a sAC gene (*ADCY10* in mammals), do not have *SLC9C* or *CatSper*. On the other hand, the presence of *SLC9B* does not predict *CatSper* gene conservation. The authors suggest that this phylogenetic data is evidence that sAC, SLC9C, and CatSper are functionally intertwined [47].

When mouse sperm are treated with 5-(*N*,*N*-dimethyl)-amiloride (DMA), a potent inhibitor of NHEs, the cytoplasm acidifies and motility is reduced [35]. DMA was found not to affect KSper or CatSper current directly, but patch clamp recordings found that DMA application resulted in both KSper and CatSper inhibition (when extracellular pH was at physiological levels and the pipette solutions were not buffered), leading to depolarization of the membrane potential and decreased intracellular Ca^2+^ levels. When pipette solutions were buffered, DMA did not have this effect, suggesting that the observed KSper and CatSper inhibition were direct results of the acidification due to NHE(s) inhibition. These results suggest that NHE activity is responsible for modulating proper KSper and CatSper activity under physiological conditions [35]. However, it is currently unclear which NHE(s) are being inhibited by DMA, therefore repeating these experiments with different NHE knockout mouse sperm could help uncover the specific NHE(s) involved. It has been noted that CatSper activity is unaffected in NHE10 KO mice [160] however, these experiments were performed with buffered pipette solutions, which would minimize any pH_i_ decrease that the NHE10 loss may have caused in the mouse sperm, leaving open the possibility that NHE10 activity helps modulate CatSper activity in mouse sperm [35].

### 10.4. NHEs versus Hv1 Ion Channel: Determining the Regulator of pH_i_ Human Sperm

The voltage-sensitive proton channel Hv1 is expressed at a high concentration in the principal piece of human sperm, and has been proposed to be the main membrane protein responsible for alkalizing these cells [161,162]. Hv1 only allows protons out of human sperm and Hv1 currents activate when the sperm membrane potential is set to 0 mV or greater under physiological pH conditions [161]. In addition, structural studies found that the Hv1 channel is open under depolarizing conditions and closed under hyperpolarized conditions [163]. Human sperm express both a full length Hv1 protein and a truncated Hv1 termed Hv1Sper, which is a post-translationally cleaved version of Hv1 missing the first 68 amino acids of the *N*-terminus [151]. Hv1Sper has reduced pH sensing and lower (less positive) activation currents (V_1/2_) than Hv1. The Hv1 proton channel is not expressed in mouse sperm, which is likely why Hv1 knockout male mice are fertile [164,165]. Of note, studies suggesting that Hv1 is the major pH regulator use patch clamping of the sperm, which is only able to measure electrogenic currents, and therefore would not detect the activity of any electroneutral NHEs that are regulated by voltage.

Hv1 has recently been shown to be directly activated by albumin binding, and while the albumin concentration in human semen is too low to activate Hv1, the higher albumin concentration in the female reproductive tract is high enough to activate it [166]. Albumin binding shifts the activation threshold of Hv1 to a more negative physiologically relevant V_m_, which helps explains how Hv1 could contribute to human sperm alkalization during the capacitation process [166]. Supporting its importance, inhibition of Hv1 results in a decrease in the percent of capacitated human sperm that were able to undergo the acrosome reaction and decreased CatSper-mediated Ca^2+^ influx [167]. All of this is strong evidence that efflux of H^+^ via Hv1 plays an important role in the sperm capacitation process in humans, however, how the activity of NHEs fit in with Hv1 activity in human sperm physiology are still incompletely defined and an important future area of study.

Ionic conditions in the female reproductive tract seem to suggest an important role for NHE activity in sperm physiology; as the sperm exit the cauda epididymis, the [Na^+^] is <25 mM and the pH is <7 however as the sperm travel through the female reproductive tract, they encounter a higher [Na^+^] and more alkaline conditions [168]. In addition, capacitated human sperm have decreased pH_i_ when incubated in media that lacks extracellular Na^+^ [40,41], suggesting a role of NHE(s) in pH_i_ regulation of sperm. The human sperm head and principal piece, but apparently not the midpiece, alkalize in response to capacitation [21]. Based on their locations, this suggests that NHE1 and NHE5 may be responsible for maintaining basal pH regulation in the midpiece, while activation of NHE10, NHA1, or Hv1/Hv1Sper is responsible for alkalization in the principal piece. Since the sperm E_m_ hyperpolarizes after capacitation, it is likely that increases in NHE10 activity is responsible for the observed increased alkalization in the principal piece, at least in mice [84]. However, NHA1 activity has been shown to require a hyperpolarized E_m_ for activation in response to zona pellucida proteins [11], and therefore NHA1 may be playing a role similar to the role proposed for NHE10 in the principal piece. NHE11 is the only known NHE to localize to the head of mammalian sperm, and therefore it may be involved in regulating pH_i_ in the sperm head in response to capacitation.

The NHE inhibitor EIPA is able to inhibit pH_i_ regulation in capacitated human sperm [40], although it is unclear which NHE(s) EIPA inhibits in human sperm. EIPA has been shown to inhibit NHE1, and to a lesser degree NHE5, but it is unclear yet if EIPA inhibits mammalian NHA1, NHA2, NHE10, or NHE11 [7,70,125,142]. Interestingly, EIPA did not affect the ability of capacitated human sperm to undergo the acrosome reaction by progesterone stimulation, suggesting that EIPA-sensitive NHE activity is not essential for the acrosome reaction [40]. Knowing the relative contribution of each NHE to regulating human sperm pH_i_ during different physiological processes is important to understanding the specialized functional roles of NHEs and Hv1; it is likely that multiple NHEs as well as Hv1 perform different functions in human sperm physiology, all of which are important for male fertility.

Interestingly, cell culture studies support the idea that Hv1 and NHE10 may have different cellular roles. Due to technical difficulties, it was necessary to measure NHE exchange in reverse in order to determine whether the VSD of the suSLC9C protein regulates its NHE activity. To overcome this, human Hv1 was co-transfected into the CHO cells and then NHE activity was measured in reverse exchange mode so that the suSLC9C protein would acidify the cell under hyperpolarized V_m_ conditions and then Hv1 would restore the original pH_i_ under depolarized V_m_ conditions. Therefore, when expressed in transfected cells, suSLC9C and human Hv1 are not active at the same V_m_ [81], suggesting potential functional differences for sperm physiology. However, because these experiments were performed in cell culture, applying these results to sperm cell physiology must be undertaken cautiously. In support of this theory, a recent analysis of pH regulation in human sperm suggests that Hv1 does not function as the main alkalizing agent in human sperm, but rather functions to buffer acidification that occurs during the generation of HCO_3_^−^ from the CO_2_ that diffuses into the sperm cell, which is why it is important for capacitation [89]. This study also suggests that amiloride-sensitive NHE exchange is responsible for establishing the resting pH_i_ regulation of human sperm as amiloride completely blocked alkalization normally observed during the transfer from acidic to alkaline conditions (as would be observed physiologically during the passage of sperm from the acidic epididymis to a more alkaline oviduct environment), while Zn^2+^ (a potent inhibitor of Hv1) had no effect on the alkalization. Additional experiments found that H^+^ efflux and Na^+^ influx under these conditions were not significantly different, suggesting that these processes are coupled, demonstrating further support of NHE activity in the alkalization of human sperm in this transition from an acidic to a more alkaline environment. It should be noted though that the identity of the specific NHE(s) responsible is/are still unknown [89]. This is further evidence that both Hv1 and NHEs are important for pH regulation in human sperm, although they are likely performing independent functions.

### 10.5. NHEs and the Functional Dependence on Na,K-ATPases

NHEs are thought to rely on the electrochemical gradient established by the Na,K-ATPase in sperm [51], and therefore the differential localization of the individual NHE isoforms and Na,K-ATPase alpha isoforms may suggest more specific functional dependence between individual isoforms of these proteins. Mammalian sperm express two different alpha Na,K-ATPase subunit isoforms (the alpha subunit is the enzymatically active subunit of the Na,K-ATPase), the alpha1 isoform and the alpha4 isoform. The alpha1 isoform localizes to the entire sperm flagellum in both rat and human [169,170]. The alpha4 isoform localizes to the midpiece of the sperm flagellum in rats [51,171] and has been reported to localize in human sperm to the principal piece [172], head and flagellum [173], and midpiece [170]. While there seems to be some discrepancy as to which specific subsection(s) the alpha4 isoform localizes to, it is clearly in the sperm flagellum. It would be interesting to see if either alpha1 or alpha4 differentially coimmunoprecipitate with any specific NHE isoforms in sperm, as this could suggest a specialized complex of functionally coupled transporters. Loss of the alpha4 isoform in mice results in male infertility due to abnormal Na^+^ regulation and depolarized membrane potential [174]. Plasma membrane alpha4 expression and activity were found to increase during capacitation in rat sperm [175]; alpha4 activity provides the Na^+^ gradient for NHE exchange while also hyperpolarizing the E_m_, potentially setting the electrical gradient necessary for voltage sensitive NHEs. Future studies into the role of both the alpha1 and alpha4 Na,K-ATPase isoforms are necessary to examine their functional significance to NHEs in sperm. It should be noted that, since the transport activity of every NHE found in sperm has not been characterized, it is possible that some of them may not function as conventional NHEs (importing Na^+^ and exporting H^+^) and therefore may not rely on the electrochemical gradient established by Na,K-ATPase activity.

### 10.6. Importance of the Characterization of NHE Transport Activity in Sperm

To fully understand the contribution of each NHE to sperm physiology, it is imperative to characterize the NHE transport activity of each NHE in these cells. Although the transport activity of some NHEs have been characterized in vitro, it is possible (and highly likely), that these proteins exhibit different transport activity in sperm than they do when expressed in cultured cells. Therefore, it is of importance to characterize the activity of these NHEs in in vitro and in vivo assays, using combinations of heterologous expression in cultured cells, reconstitution of purified proteins in defined lipid environments, and NHE knockout mouse sperm models. Interestingly, out of all of the NHEs expressed in mammalian sperm, the only three that have yet to have their transport activities characterized to at least some degree in heterologous cell culture systems are those that display testis/sperm specific expression (NHA1, NHE10, and NHE11). Our experience suggests that it is extremely difficult to express these three NHEs in cell culture using known methodologies. In support of our observations, a recent study found that the VSD of NHE10 is highly toxic to both bacteria and cultured mammalian cells [176]. Thus, an effort to make novel gene-edited mouse models, such as those containing point mutations that would alter amino acids thought to be important for transport function, to characterize these proteins is essential to fully understand how each NHE contributes to sperm physiology.

In addition, NHE proteins are known to interact with and be modulated by several accessory proteins. Therefore, in the various NHE KO mouse models that exhibit male fertility defects, it is important to determine whether it is the loss of NHE transport activity that is responsible for the observed male infertility, or if it the loss/dysfunction of proteins that associate with that NHE that is responsible for the observed phenotype.

### 10.7. NHEs as an Attractive Target for Male Contraceptive Drugs

Since various NHEs have been shown to be essential for male fertility in mice and humans [8,9,10,11,12,13,14,15], NHEs have been posited as a possible target to design male contraceptive drugs against. However, some of the NHEs that are essential for male fertility in mice, NHE3 and NHE8 [9,14,15,42,71,149], also exhibit high expression levels in somatic tissues and therefore a specific inhibitor of these isoforms is likely to have unintended negative consequences in other tissues. Therefore, the more enticing targets are the NHEs that exhibit testis/sperm-specific expression such as NHE10, NHE11 and NHA1. Out of these three NHE isoforms, NHE10 is likely the most promising as there is functional data suggesting that NHE10 protein expression is essential for male fertility in both mouse and humans [8,13]. On the other hand, there is currently no functional data regarding whether NHE11 is necessary for mammalian male fertility. While NHA1 is important for male fertility in mice [10,11], designing a specific inhibitor of NHA1 without inhibiting the ubiquitously expressed NHA2 protein may be difficult as the NHE domains of these two transporters are highly conserved [73,77]. Additionally, as noted above, characterization of the transport activity of NHE10, NHE11 and NHA1 is necessary to determine if it is loss of NHE activity or loss of other proteins that associate with these NHEs that results in the infertility phenotype, as this information will inform which part of the NHE to target.

### 10.8. Models for NHE Contribution to Mammalian Sperm Physiology

**Figure 5:**
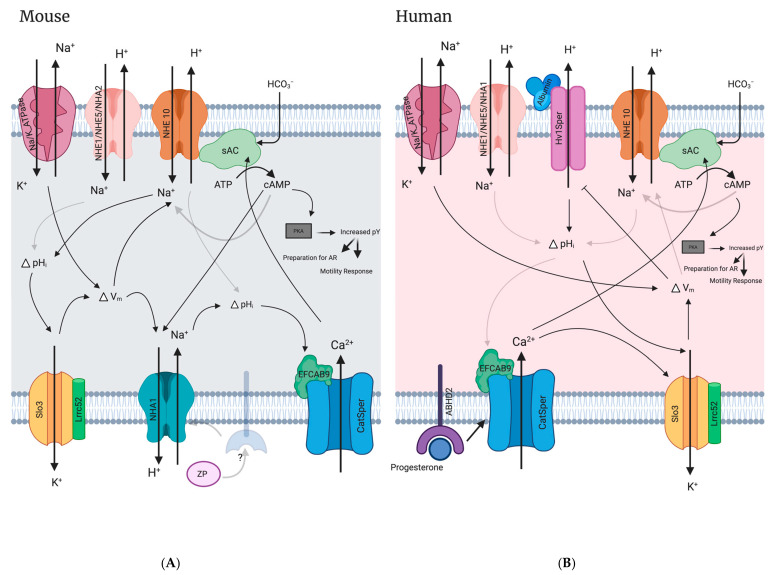
Signaling pathways in mammalian sperm flagellum. (**A**) Mouse sperm flagellum signaling pathways involving NHEs. ZP; zona pellucida protein. (**B**) Human sperm flagellum signaling involving NHEs. See main text (Section 10.8) for detailed descriptions of pathways. Solid arrows indicate literature validated mechanisms. Faded arrows indicate proposed or putative mechanisms. pY; phosphorylated-tyrosine protein levels. Figure created with Biorender.com.

While we recognize that many aspects of the intricate signaling pathways remain to be elucidated, especially in human sperm, we present here a potential working model for NHE activity in mammalian sperm physiology (Figure 5). Immediately following ejaculation into the vagina, various NHEs in the flagellum and potentially Hv1/Hv1Sper (in the principal piece of human sperm) participate in maintaining a basal pH under non-capacitating conditions. As sperm leave the vagina and enter the cervix to continue along the reproductive tract, they are exposed to signaling molecules and changing ionic concentrations which favor capacitation. As the sperm encounter a higher extracellular Na^+^ concentration, basal NHE activity (potentially due to multiple NHEs) in the midpiece and principal piece increases. This increase in sperm intracellular pH (as well as exposure to an increasingly alkaline pH environment in the female reproductive tract) activates Slo3 in mouse sperm which causes an efflux of K^+^ and hyperpolarization of the sperm E_m_. The hyperpolarization of the E_m_ via Slo3 is necessary in mouse sperm prior to CatSper activation, but in human sperm, SLO3 is strongly activated by Ca^2+^; progesterone-activated CatSper activity is enough to activate SLO3. Therefore, it is currently unclear what the order of SLO3 and CatSper activation in human sperm is. In mouse sperm, the hyperpolarization caused by Slo3 activation activates NHE10 activity, which causes an increase in pH_i_, further activating Slo3, therefore, these two proteins act to positively regulate each other once one is activated. This mutual NHE10-SLO3 activation can explain two hallmarks of sperm capacitation: sperm membrane hyperpolarization and an increase in sperm pH_i_. All the while, Na,K-ATPases are continuously active in order to establish and maintain the Na^+^ and K^+^ gradients used by NHE10 and Slo3. The increase in pH_i_, due to NHE activity, activates CatSper, resulting in transient Ca^2+^ influx which finely controls capacitation associated events such as sAC synthesis of cAMP-sAC activity which is activated both by Ca^2+^ and HCO_3_^−^. The increase in cAMP leads to activation of PKA and sets off a signaling cascade ultimately resulting in an increase in phosphorylated tyrosine residues (pY) in sperm proteins, a hallmark of sperm capacitation. The sperm hyperpolarization and increase in intracellular cAMP achieved in capacitation acts to prime mouse sperm for exposure to zona pellucida proteins from the oocyte. The zona pellucida proteins activate NHA1 to further alkalize the cell, again activating CatSper to mediate Ca^2+^ influx and initiate additional signaling events for fertilization of the egg. The exact start of capacitation events in human sperm and the role that NHEs play is more enigmatic because human SLO3 is activated more strongly by Ca^2+^ than pH_i_. Therefore, it is possible that CatSper activation via progesterone (released from the oocyte) precedes SLO3 and NHE10 activation in human sperm. It is also unclear whether NHA1 plays the same role in zona pellucida induced alkalization in humans as it does in mice.

## 11. Conclusions

NHEs are important for male fertility in many species, spanning sea urchins to humans. There are currently eight known NHEs that are expressed in mammalian testis/sperm, however the exact role of each of these NHEs in male fertility remains to be determined. Future work is needed to characterize the transport activity of all of these NHEs in a testis/sperm-specific context. Of particular interest is further characterization of the human NHE10 and NHE11 proteins because of their potential regulation by voltage and cyclic nucleotides which are known to be important for capacitated sperm and NHE10 has also been shown to be essential for male fertility. NHE10 has been shown to be essential for human male fertility and exhibits testis/sperm-specific expression and is therefore an attractive target for male contraceptive drugs. An exciting area of future research, NHE11 specifically localizes to the sperm head, the only NHE known to do so in rodent and human sperm. Although its contribution to male fertility is unknown, NHE11 transport activity is also likely regulated by E_m_ and cyclic nucleotides, and its unique localization positions it to potentially regulate important sperm physiology events such as the acrosome reaction or sperm-egg fusion/fertilization events. Finally, investigating all of the NHEs expressed in sperm will give us a better understanding of the intricacies of ion transport and regulation involved in male reproductive biology as well potentially identifying abnormalities that affect some human males currently suffering from infertility.

## Figures and Tables

**Table 1:** Characteristics of the NHEs expressed in mammalian sperm. * Sea urchin SLC9C1 transport activity data is listed for the biochemical activity of NHE10 and NHE11 because no mammalian data exists yet.

Gene (Protein)	Tissue Expression	Subcellular Localization	Sperm Localization	Transport Activity	Mouse Male Fertility KO Phenotype	Human Male Fertility Phenotype	Known Inhibitors	References
***SLC9A1* (NHE1)**	Near ubiquitous	Plasma membrane	Midpiece	Na^+^/H^+^ 1:1 (electroneutral)	Fertile	Unknown	Amiloride, EIPA, HMA, DMA, HOE-694, SM-20550, S-3226, clonidine, cimetidine	[7,50,51,58,59,60,61]
***SLC9A3* (NHE3)**	Highly expressed in digestive tract and kidney; low expression in testis and other	Plasma membrane and endosomal (recycling)	Developing acrosome	Na^+^/H^+^ 1:1 (electroneutral)	Sperm lack acrosomes; Vas deferens defects; Infertile	Congenital bilateral absence of the vas deferens (CBAVD); Infertile	Amiloride, tenapanor, HMA, DMA, HOE-694, zoniporide, S-3226, clonidine, cimetidine	[6,15,42,59,62,63,64,65,66,67]
***SLC9A5* (NHE5)**	Brain, kidney, testis, other	Plasma membrane and endosomal (recycling)	Midpiece	Na^+^/H^+^ 1:1 (electroneutral)	Fertile	Unknown	Amiloride, EIPA, HMA, HOE-694, cimetidine	[51,59,68,69]
***SLC9A8* (NHE8)**	Ubiquitous	Intracellular organelles	Developing acrosome	Na^+^/H^+^ (electroneutral)	Sperm lack acrosomes; Infertile	Unknown	HOE-642, S-3226	[9,16,60,70,71]
***SLC9B1* (NHA1)**	Testis/Sperm specific	Plasma membrane	Principal piece	(Likely based off of structural homology models) Na^+^/H^+^ (electroneutral)	Submotile sperm, subfertile; NHA1/2 dKO completely infertile	Unknown	Unknown	[10,11,72,73]
***SLC9B2* (NHA2)**	Near ubiquitous	Plasma membrane; Intracellular (cell type specific)	Principal piece	Na^+^(Li^+^)/H^+^ (electroneutral)	Submotile sperm, subfertile; NHA1/2 dKO completely infertile	Unknown	Phloretin	[10,74,75,76,77,78,79]
***SLC9C1* (NHE10)**	Testis/Sperm specific	Plasma membrane	Principal piece; Flagellum	Unknown * Na^+^/H^+^ Exchanger 1:1 (electroneutral); Voltage sensitive; Cyclic nucleotide gated	Immotile sperm; Infertile	Immotile sperm; Sperm structural defects; Infertile	Unknown	[8,13,80,81]
***SLC9C2* (NHE11)**	Testis/Sperm specific	Likely plasma membrane	Sperm head (likely plasma membrane overlaying acrosome)	Unknown * Na^+^/H^+^ Exchanger 1:1 (electroneutral); Voltage sensitive; Cyclic nucleotide gated	Unknown	Unknown	Unknown	[6,43,81]

## Data Availability

No new data were created or analyzed in this study. Data sharing is not applicable to this article.
